# CD40 monoclonal antibody and OK432 synergistically promote the activation of dendritic cells in immunotherapy

**DOI:** 10.1186/s12935-022-02630-x

**Published:** 2022-06-17

**Authors:** Juan Zhang, Lei Wang, Shuyi Li, Xuefeng Gao, Zhong Liu

**Affiliations:** 1 Department of General Surgery, Shenzhen University General Hospital, Shenzhen University Clinical Medical Academy, Shenzhen, Guangdong China; 2 Central Laboratory, Shenzhen Key Laboratory of Precision Medicine for Hematological Malignancies, Shenzhen University General Hospital, Shenzhen, Guangdong China; 3 Department of Hematology and Oncology, International Cancer Center, Shenzhen Key Laboratory, Shenzhen University General Hospital, Shenzhen University Clinical Medical Academy, Shenzhen University Health Science Center, Shenzhen, Guangdong China; 4 Department of Radiation Oncology, Fujian Cancer Hospital, Fujian Medical University Cancer Hospital, Fuzhou, Fujian China

**Keywords:** Colorectal cancer, CD40 mAb, Cytotoxic T lymphocytes, OK-432, Pulmonary metastasis

## Abstract

**Background:**

Colorectal cancer (CRC) with pulmonary metastasis usually indicates a poor prognosis, whereas patients may benefit from adoptive cell therapy. Tumor-specific cytotoxic T lymphocytes (CTLs) have been reported as a promising treatment for CRC. However, the antitumor effect of CTLs remains limited partially due to insufficient production of effector cells via the activation by antigen-presenting dendritic cells (DCs).

**Method:**

This study showed that a combination of CD40 mAb and Picibanil (OK-432) could significantly enhance the activation of CTLs by DCs, both in vitro and in vivo. Flow cytometry, colon cancer mouse model, and pathological staining were employed to demonstrate the specific functions.

**Results:**

This approach promoted the maturation of DCs, augmented the production of stimulatory cytokines, and suppressed the secretion of inhibitory cytokines. Additionally, it facilitated the killing efficiency of CTLs via stimulating their proliferation while restraining the number of Tregs, concomitantly with the positive regulation of corresponding cytokines. Furthermore, the combined unit could hurdle the expansion of tumor cells on metastatic lungs in the colon cancer mouse model.

**Conclusion:**

Collectively, the combination of CD40-mAb and OK-432 facilitated the maturation of DCs and enhanced the cytotoxicity of T cells, promising therapeutic approach against CRC.

**Graphical Abstract:**

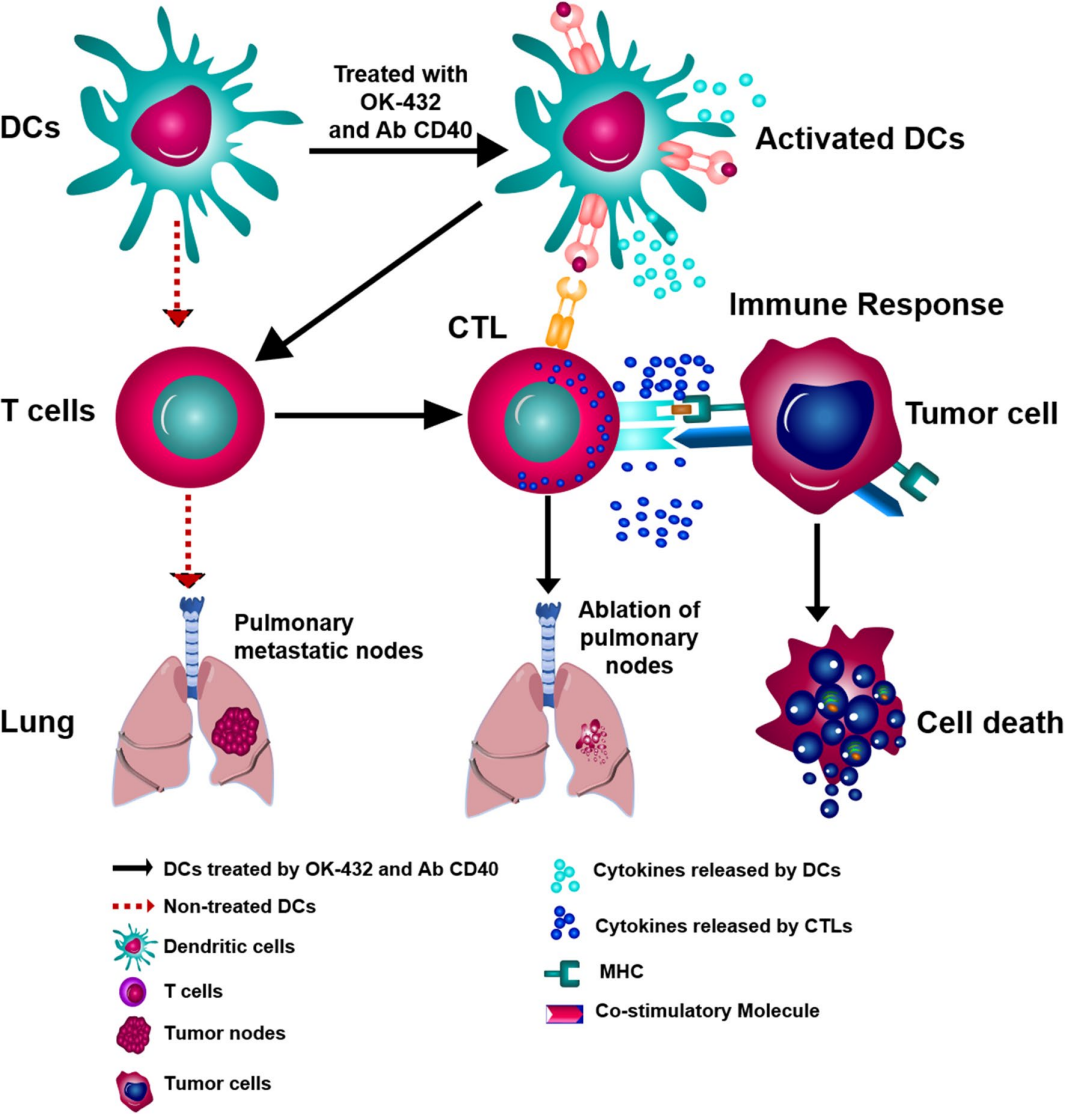

**Supplementary Information:**

The online version contains supplementary material available at 10.1186/s12935-022-02630-x.

## Introduction

Immunotherapy has shown a promising treatment for the advanced gastrointestinal malignancies. However, the survival rates of patients with advanced colorectal cancer (CRC) remain very poor [1]. Therefore, more effective approaches against metastatic CRC (mCRC) are urgently needed. Based on the crucial components including cytotoxic T cells, suppressive regulatory T cells, and stimulatory and inhibitory cytokines, the modified strategy against CRC should be performed to boost the beneficial events and suppress the inferiors [2].

As a human defensive system, the immune system is furnished with innate and adaptive immunity to surveillant unnatural cell growth to prevent cancer progression [3, 4]. However, the components involved in immune system often compromised their immune response by tumor escape mechanisms, including MHC downregulation, antigen hiding, and checkpoint ligand expression [4, 5].Targeting such problem, scientists have developed various approaches to boost the compromised immune cells with cytotoxicity [6]. Immunotherapy, as one of strategies, has powerfully altered the landscape of malignancy treatment, including mCRC. It has exhibited improved antitumor immunity and tremendous potential for reversing the immunosuppression induced by tumors [5]. For instance, immune checkpoint inhibitors (ICIs) have led the immunotherapy field to regain a major influence on clinical efficacy in patients with tumors [7]. However, immune-related adverse events are inevitable, as ICIs usually elicit long-lasting clinical responses via reactivating an exhausted immune response. Adoptive cell therapy (ACT) therapy including CAR-T, TCR-T, CTL, CIK, and NK have shown promise in cancer-targeting immune infiltrate and specific immune response [5]. It can provide long-term durability in solid tumors, which is unachievable for chemotherapy [8, 9]. Patients with metastatic cancer have obtained inspired clinical outcomes through ACT therapy. For instance, CIK was used to target renal, hepatocellular, and nasopharyngeal carcinoma with limited graft-versus-host effects; CTLs show efficacy on metastatic melanoma; CAR-T is beneficial to refractory lymphoma; and TCR-T is employed to target MART-1 for the treatment of metastatic melanoma [10–12].

Dendritic cells (DCs) play a vital role in the immune response, especially the stimulation of cytotoxic T lymphocytes (CTLs). Landmark studies have demonstrated that CD8^+^ CTL density is strongly associated with disease-free and overall survival in patients with CRC, which has been employed as a prognostic biomarker for the survival of such patients [13, 14]. ACT has displayed powerful potential in the development of CRC treatment in the last decades. CTL-mediated inflammatory response to tumor cells is critical in suppressing the progression of carcinogenesis [15]. Therefore, approaches improving the generation of CTLs are vital for the immunotherapy of patients with CRC.

The cytotoxicity of CTLs critically depends on DCs [16]. As professional antigen-presenting cells (APCs), DCs play essential roles in both innate and adaptive immunity systems upon pathogen invasion [17]. Immature DCs take up antigens through phagocytosis, micro- or macro-pinocytosis, and endocytosis using Fc receptors, integrins, C-type lectin receptors, apoptotic cell receptors, and scavenger receptors. The mature DCs can transport cancer-associated antigens to the draining lymph nodes, where T cell priming and activation can occur to generate CTLs [18]. Various studies have shown that CTLs induced by DCs can enhance the antitumor effect against CRC [19–22]. Therefore, targeting DCs may confer a feasible strategy for CRC immunotherapy. The inhibitory cytokines (IL-10 and TGF-β) and stimulatory cytokines (IL-12 and IFN-γ) play opposite roles during CTL generation [23, 24]. The former is involved in the suppression of T cells, whereas the latter stimulates them. Produced by Tregs, IL-10 and TGF-β majorly play a suppressive role in the immune system. As a pro-inflammatory cytokine, IL-12 be produced by mature DCs upon pathogen infection, which contributes to the activation of T cells and natural killer (NK) cells and the secretion of IFN-γ that favors the differentiation of Th1 cells. Additionally, it helps to forms a link between innate resistance and adaptive immune. Besides, IL-12 can suppress the proliferation of Tregs and the Foxp3 level, favoring the outgrowth of non-Tregs [25]. Conferred with the diverse capability, IL-12 is usually employed as an adjuvant of ACT therapy, which can boost many direct and indirect antitumor activities correlating with innate and adaptive immunity. A phase I study reported that recombinant human IL-12 showed significant immunomodulatory effects in 20 patients with renal cell cancer and 12 patients with melanoma. However, later clinical studies with IL-12 did not yield satisfactory results [26, 27]. Collectively, IL-12 and IFN-γ majorly function as a stimulator favoring the activation of cytotoxic cells. Furthermore, DCs themselves also act as direct killers targeting tumor cells and induce the dying cells to release tumor-related antigens [28].

A successful adaptive immune response required not only primary signals (binding of receptor and ligand) but also multiple secondary signals, among of which CD40 acts as an essential co-stimulator [29]. As a cell-surface member of the tumor necrosis factor (TNF) receptor superfamily, CD40 is expressed on a variety of APCs. As a costimulatory molecule, it is vital for activating APCs and CD8^+^ T cells [30]. The binding of CD40 and CD40L not only augments the B cell activation and proliferation but also promotes the maturation of APCs, such as enhancement of MHC II expression and CD80/CD86 upregulation [31]. The CD40–CD40L signaling plays a central role in the regulation of the immune response through an interaction between T cells and APCs. It can assist antigen presentation and promote the expression of costimulatory molecules, which allows the maturation of DCs and stimulation of T cells. Upon activation of lymphocytes, the binding of CD40L on the surface of T cells with CD40 provides costimulatory molecules required to activate naive T cells, thereby amplifying the immune response [32]. Study also reported that CD40-CD40L pathway is required in the process of tumor elimination mediated by nitric oxide synthase. The survival of CRC patients is strongly correlative with nitric oxide synthase, CD40, and TNF expression [33]. Besides, CD40 is widely expressed by various tumor types including CRC. It has been employed as prognostic biomarker and a potential target for immunotherapy [34]. There is a correlation between CD40 expression and CRC differentiation. Researchers showed that the signaling of CD40-CD40L binding can inhibit the CRC proliferation and induce apoptosis [35]. Additionally, the CD40L level of CRC patient is significantly associated with thrombocytosis, IL-6, and emergence of distant metastases. The high level of CD40L is correlated with more advanced CRC with worse prognosis [36].

Preclinical investigations showed that CD40-activated DCs were poised to prime or activate tumor-specific T cells [32]. Moreover, CD40 activated macrophages were shown to infiltrate tumors and destroy tumor stroma via tumoricidal activity [37]. Besides, as a membrane antigen, CD40 can regulate the production of costimulatory cytokine IL-12. These data inspired the use of CD40 agonists, particularly the CD40 monoclonal antibodies (mAb), as a novel approach for cancer immunotherapy. It has shown feasibility in both preclinical and clinical settings [30]. Evidences suggest that the activation of CD40 is critical in the conversion of “cold tumor” into “hot tumor,” which makes the tumor more susceptible to ICIs [38]. In the last few years, CD40 mAb combined with other therapies has shown remarkable efficacy, which is potentially important to extend the effective range of CD40 mAb. CD40 antibodies with various formulations have shown tolerable and feasible effects in patients with mesothelioma, pancreatic cancer, and other cancer types, when combined with chemotherapy [39]. Their combination may codetermine the terminal outcomes, which needs more attention. Although CD40 mAb is important for immunotherapy, it has some deficiencies to be complemented; CD40 mAb simultaneously stimulates the production of stimulatory cytokine IL-12 and the inhibitory cytokine IL-10 [40]. Therefore, exploring an adjuvant to neutralize the side effects of CD40 mAb is necessary.

OK-432 is a lyophilized preparation from penicillin-inactivated *Streptococcus pyogenes* [41]. Mature DCs can produce pro-inflammatory cytokines, which are beneficial to the proliferation of CTLs. This cytotoxicity is mediated by the CD40/CD40 ligand axis, as CD40 ligand on DCs can interact with CD40 on tumor cells. Studies reported that OK-432 can activate human DCs via CD40-CD40 ligand interactions [42, 43]. The effect of OK-432 on DCs may be achieved via the peptide specific: inducing the generation of IL-12 and IFN-γ; upregulating the expression of CD40, CD80, CD86, adhesion molecules (ICAM-1), and so forth; and stimulate the antigen-specific cytotoxic lymphocytes. These data endowed OK-432 with the potential in anticancer immunotherapy, indicating a feasible clinical application approved by clinical observation in CRC [44, 45].

Based on the aforementioned evidence, we proposed that the combination of CD40 mAb and OK-432 might confer a synergistic effect on the activation of DCs, which determined the generation of CTLs. We employed a mouse model of CRC lung metastasis to demonstrate the therapeutic effect of this combination. Briefly, we first observed that OK-432 contributed to the activation of DCs, which is via CD40/CD40 ligand pathway. Meanwhile, CD40–CD40L signaling plays a central role in the regulation of the immune response through an interaction between T cells and APCs. It can assist antigen presentation and promote the expression of costimulatory molecules, which allows the maturation of DCs and stimulation of T cells. Our results showed that the addition of CD40 Ab stimulates CD40 receptor, which collaborates with OK-432 to boost the immune response including the activation of DCs, stimulation of cytotoxic T cells, suppression of regulatory T cells, and releasing of beneficial cytokines. Collectively, we summarize the study in graphic abstract. In normal condition, dendritic cells can activate antigen-specific T cells, which then transform into cytotoxic T lymphocytes (CTLs). They are conferred with cytotoxicity against tumor cells. We employed the combination of OK-432 and CD40 Ab as a booster of DCs. The study shows that such combination augments the activity of DCs, including upregulation of CD80/CD86 receptors, increased releasing of cytokines. The boosted DCs can subsequently stimulate T cells to more proliferation, tighter binding with tumor, more releasing of cytokines. The CTLs induced by boosted DCs are conferred with stronger cytotoxic to tumor cells and metastatic tumor in lung, leading to tumor cell apoptosis and metastatic tumor collapse.

## Materials and methods

### Reagents and cell lines

OK-432 was purchased from T&L Biological Technology (GMP-TL107-0100). CD40 mAb was purchased from AdipoGen (AG-20B-0036PF). Antibodies used in the FCM were purchased from Miltenyi Biotec, and are listed in Additional file 1: Table S1. The information of cytokines used in the experiment is also provided in Additional file 1: Table S1. Human colorectal cancer SW-620 cells and mouse colon cancer CT26 cells were obtained from the Institute of General Surgery, Chinese PLA General Hospital (Beijing, China). SW-620 and CT26 cells were cultured in Dulbecco's modified Eagle's medium (Gibco, 11,965,092), supplemented with 10% heat-inactivated fetal bovine serum (Gibco, 16,000,044) and 100 × penicillin–streptomycin solution (Corning, 30-002-CI, USA) at 37 °C in a 5% CO_2_ incubator (Thermo Scientific, MA, USA). When the cells reached more than 90% confluence, they were detached using 0.25% trypsin (Gibco, 25,200,056) and passaged at a ratio of 1:4.

### Ethics statement

The experimental mice used in this study were obtained from the Laboratory Animal Center of the Academy of Military Medical Science. All experimental and postoperative animal care procedures were performed according to the protocols approved by the Animal Care and Use Committee of the Chinese PLA General Hospital and the National Institute of Health Guidelines for the Care and Use of Laboratory Animals. As the experimental performance was relatively harmless, we did not use anesthetics until the last step of sacrifice, which was terminated by CO_2_ narcosis performed by trained personnel. According to the PACUC guideline, the mice were displaced in a 10-L volume chamber (less than five mice per cage), and compressed CO_2_ gas in cylinders was used at a flow rate of 5 L/min (50% of the chamber volume), lasting for 2–3 min until the mice were unconscious. Subsequently, cervical dislocation was performed.

The study on clinical samples was conducted according to the guidelines of the Declaration of Helsinki and approved by the ethics committee of the First Affiliated Hospital of Dalian Medical University (Dalian, Liaoning, China). A written informed consent was obtained from each subject following the Declaration of Helsinki.

### Isolation of human DCs

Human DCs were collected as described in a previous study [46]. Generally, PBMCs were isolated from the freshly prepared whole blood. Firstly, aliquot 20 mL whole blood into 50 mL conical tube and diluted it at 1:1 with RPMI-1640 medium. Underneath the blood-PBS mixture, 10 mL Ficoll–Hypaque was layered slowly. The mixture was then centrifuged at 500 ×*g* for 20 min at RT (without braking). The mononuclear cell layer was collected and aliquoted 20 mL, which was then transferred into 50 mL conical tube, filled with cold running buffer to 50 mL. Spin down the mixture at 300 ×*g* for 10 min at 4 °C, depleting the platelets. Pellet was rinsed once and collected at 500 ×*g* for 5 min at 4 °C, which was then resuspended in a 50 mL tube at 1 × 10^8^ MNCs/mL. The MNCs were then mixed with non-DC cells antibody cocktail (Human DC Enrichment Kit, ThermoFisher Scientific, 11308D) and incubated for 20 min at 2–8 °C. Spin down at 300 ×*g* for 10 min at 2–8 °C. Rinse the pellet and incubate it with the pre-washed dynabeads 15 min at 2–8 °C. The magnet was used to deplete non-DC cells. DC population was the negative fraction involved in the supernatant and could be used for subsequent experiments.

### Isolation of mouse bone marrow DCs

The bone marrow DCs (BMDCs) were collected as described in a previous study [47]. Briefly, intact femur bones were isolated from 6-week-old BALB/c mice and sterilized in 75% ethanol for 5–10 s. Scissors were used to cut both ends of the isolated bones. Syringes filled with HBSS were used to flush out the bone marrow from each bone until it turned to white. The bone marrow suspension was then collected and spin down at 250 g for 10 min. Resuspended the pellet at a final density of 10 × 10^6^ cells/mL for subsequent seeding. The cells were cultured in regular culture medium (RPMI-1640 + 10% FBS + 20 mM penicillin/streptomycin) at a final cell density of 2 × 10^6^ cells/ml, supplemented with 20 ng/mL rmGM-CSF for 6–10 days in a 37 °C incubator with 5% CO_2_, during which the medium with rmGM-CSF was refreshed every 3 days. Then, the non-adherent and loosely adherent cells in the culture supernatant were harvested rinsed by PBS. Resuspend the pellet among which anti-F4/80^+^ magnetic beads were employed to deplete the macrophages (marked by F4/80^+^ and CD11c^+^). Thereby, DCs (marked by CD11c^+^) were negatively selected and further purified by anti-CD11c magnetic beads, which can be used for the subsequent experiment.

### Preparation of CTLs

#### Isolation of mouse splenic T cells

The collection of splenic T cells was performed as described previously [48]. Generally, mouse spleen was freshly isolated from 6-week-old Balb/c mouse and put into cold PBS. Scissors were used to cut the spleen into fine parts. Collagenase IV (100 U/mL, Gibco, 17,104,019) and DNase (20 µg/mL, Thermo Scientific, 90,083) with 1% FBS (Gibco, 16,000,044) was employed to digest the spleen for 30 min at room temperature. Subsequently, EDTA (1 mM/mL) was used to terminate the digestion. Grind the digested tissue with flat end of a syringe, which was then filtered through a 70 μm cell strainer. Spin down (600 ×*g*) the cells and resuspended the pellet with cold RBC (red blood cell, Chinese Academy of Medical Sciences, Beijing, China) lysis buffer, which was then incubated in ice bath for 5 min. Dilute the above buffer with cold PBS and spin down (600 ×*g*) the cells. The pellet was then resuspended with complete medium (1640 medium-10% FBS). The filtered cell suspension was then resuspended with red blood cell lysis buffer (Chinese Academy of Medical Sciences, Beijing, China) to acquire the mononuclear cells. The splenic cells were then purified using a non-T-cell depletion antibody cocktail (Dynabeads^®^ Untouched™ Mouse T Cells, 11413D). The purity of T lymphocytes was checked by flow cytometry using antibodies specific for purified T cells. The T cells derived from peripheral blood were collected, as mentioned earlier, starting at the stage of red blood cell lysis.

#### Isolation of mouse splenic dendritic cells

The collection of splenic dendritic cells was performed according to the previous studies and partially modified [49, 50]. Collagenase digestion mixture was pre-prepared:1 mg/mL collagenase D (Roche, 11,088,858,001), 20 μg/mL DNase I (Thermo Scientific, 90,083), 2% FBS (Gibco, 16,000,044) in HEPES-buffered RPMI-1640 (Gibco, 11,530,586). Mouse spleen was freshly isolated from 6-week-old Balb/c mouse and put into cold PBS. Every spleen was injected with 1 ml digestion solution to make the spleen distend and change from maroon to reddish-orange. Then fragment the spleen that was then digested with 400 U/ml collagenase for 60 min at 37 °C. EDTA (1 mM/mL) was used to terminate the digestion. The digested tissue was then strained through the 70 μm cell strainer. Spin down (600 ×*g*) the cells for 5 min and resuspended the pellet with cold RBC (red blood cell, Chinese Academy of Medical Sciences, Beijing, China) lysis buffer, which was then incubated in ice bath for 5 min. Dilute the above buffer with cold PBS and spin down (600 ×*g*) the cells. The pellet was then resuspended with cold PBS. Dynabeads™ Mouse DC Enrichment Kit (Invitrogen, 11429D) was used to deplete non-DC cells. The purity of DCs was checked by flow cytometry, using anti-CD11c antibody (Invitrogen, MA5-16,877). 500 U/mL of granulocyte macrophage colony stimulating factor (GM-CSF, BD Biosciences, 554,406) and 500 U/mL of interleukin 4 (IL4, BD Biosciences, 554,433) recombinant human proteins were supplemented with the complete medium.

#### Preparation of CTLs

Conventional and autologous CTLs were prepared in our laboratory as previously reported [51, 52]. Firstly, the collected T cells were activated by CD3/CD28/CD2 antibodies (25ul/ml) for 72 h; DCs were separately treated with relative agents, which were then marked as mDCs. Then mDCs were co-cultured with T cells at a ratio of 1:10 and supplemented with 100 ng/mL CD3ε mAb (BD Biosciences, 553,057) and 10 ng /mL rm-IL-2 (BD Biosciences, 550,069). The medium with IL-2 was refreshed every other day. On the seventh day, CTLs were harvested for the subsequent experiments.

### Flow cytometer

The DCs were identified using CD11c-FITC antibody and analyzed using FACS. The subsets of DCs were sorted using corresponding antibodies such as CD8α, CD80, and CD86. The CD8α^Pos^ subset of DCs was purified using a biotin-conjugated antibody cocktail. The CD8α^Pos^ subset of DCs was then incubated with either vehicle control or positive control TNF-α, OK-432, CD40 mAb, and a combination of OK-432 and CD40 mAb.

The proportions of subtypes in T cells were detected using FCM. Briefly, the lymphocytes were first incubated with corresponding antibodies for 30 min at room temperature following the manufacturer’s protocols. For detecting Foxp3, the cells were subjected to membrane rupture and stained with Foxp3-PE (Miltenti Biotec-3G3, Cologne, Germany) following the manufacturer’s protocol. All samples were examined using a FACSCalibur instrument (Becton Dickinson, USA), and the data were analyzed using FlowJo7.6.1 software. The level of interferon-gamma (IFN-γ) in the cell culture supernatant was determined using an enzyme-linked immunosorbent assay kit (Sigma–Aldrich, MO, USA). The in vitro cytotoxicity of the CTLs (effect cell, E) to the CT26 cells (target cell, T) was assessed using an LDH release assay kit**.**

### Animal models and in vivo experiments

Six-week-old BALB/c female mice were purchased from the Beijing Experimental Animal Center of the Academy of Military Medical Sciences (Beijing, China). The colon cancer lung metastasis model was conducted as described previously [51]. Briefly, 1 × 10^5^ mouse CT26 cells in 100 μL of phosphate-buffered saline were injected into the mice via the tail vein. The administration of CTLs was performed according previous study [53]. On the fourth day, the tumor-bearing mice were randomly divided into seven groups with four mice each, including one vehicle and six treatment groups: Group 1, in which each mouse was treated with 200 μL of saline twice a week, was marked as the vehicle group; Group 2, in which the mouse was treated with CTLs (1 × 10^7^ cells) induced by DCs, was marked as the DC-CTL group; Group 3, in which the mouse was treated with CTLs (1 × 10^7^ cells) induced by DCs with anergic antigen, was marked as the Ag-DC-CTL group; and, by the analogy, group 4 to group 7 were correspondingly marked. The treatments were applied for 2 weeks. At the end of 2 weeks, the mice from each group were sacrificed to assess the antitumor effect.

### Counting of metastatic lung nodules

Counting of metastatic lung nodules and enrichment of lymphocytes from lung tissue were performed as previously described [51]. Briefly, nodule diameters of less than 0.5 mm, 0.5–1 mm, 1–2 mm, and greater than 2 mm were classified as grade I, II, III, and IV metastasis, respectively. The total numbers of metastases were calculated using the following formula: total metastasis number = (grade I metastasis number) + (grade II metastasis number × 2) + (grade III metastasis number × 3) + (grade IV metastasis number × 4). The left lung was digested, and then the mononuclear cell suspensions were collected using discontinuous density gradient centrifugation with mouse lymphocyte separation medium (MP Biomedicals, CA, USA).

### Cytotoxicity assay

The in vitro cytotoxicity of CTLs to SW-620 cells was performed using an LDH release assay (Sigma–Aldrich, MO, USA), as described previously [54]. The target SW-620 cells were mixed with CTLs derived from different treatments (vehicle control, TNF-α, CD40, OK-432, and the combination of CD40 and OK-432) at a ratio of 5:1 and cultured for 24 h. The optical density (OD) value was detected with a microplate reader at a wavelength of 492 nm. The killing rate was calculated as follows: Cytotoxicity % = [OD (Experimental) – OD (Effector spontaneous)–OD (Target spontaneous)] × 100/[OD (Target maximum) – OD (Target spontaneous)].

### Hematoxylin and eosin staining

The lungs of the control and test groups were fixed with fresh 4% formalin for 24 h at room temperature, followed by dehydration, transparent, and paraffin embedding. Then, the samples were sliced into 4-μm sections. The tissue slides were stained with hematoxylin and eosin (Biosharp, BL700A), and the histological images were observed and photographed using light microscopy.

### Statistical analysis

SPSS 21.0 statistical software was used for statistically analyze the relevant data. Data were expressed as the mean ± standard deviation. Differences between the two groups were compared using *t* tests. Differences among several groups were analyzed using one-way analysis of variance. *P* < 0.05 indicated a statistically significant difference. The survival rate was analyzed using the Kaplan–Meier method.

## Results

### CD40 mAb and OK-432 showed synergistic actions activating human DCs

**Fig. 1:**
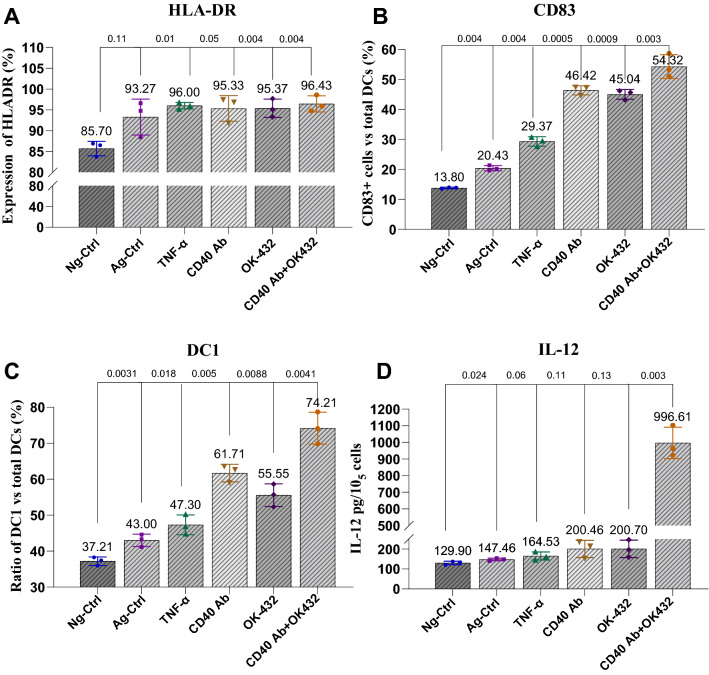
CD40 Ab and OK-432 showed synergistic function in activating human DCs. **A** Expression of HLA-DR in human DCs. The expression of HLA-DR showed a marginal increase in agent-treated groups. **B** CD40 Ab and OK-432 synergistically activated the expression of CD83. The experiment was performed in six groups, including three control groups: Neg-Ctrl (without any agent), Ag-Ctrl (with anergic antigen), and TNF-α (positive control), and three test groups: CD40 Ab, OK-432, and 40,432 groups (combinational group, CD40 Ab, and OK-432). Compared with the control groups (Neg-Ctrl, Ag-Ctrl, and TNF-α), the expression of CD83 was separately improved by CD40 Ab and OK-432, and the synergistic 40,432 group displayed the most significant improvement. **C** CD40 Ab and OK-432 synergistically improved the ratio of type 1 DCs (DC1). The number of DC1 was separately increased by CD40 Ab and OK-432; the effect in the synergistic 40,432 group was prominent. **D** Combination of CD40 Ab and OK-432 significantly promoted the production of IL-12. The secretion of inflammatory mediator IL-12 was strongly stimulated in the combinational group (CD40 Ab + OK-432). Value represents mean ± standard error of mean. P-value represents the comparison of control group versus tested groups

First, we assessed the activation effect of the combination of CD40-mAb and OK-432 on human DCs in vitro with three control groups (Neg-Ctrl: without any agent; Ag-Ctrl: 10 μg/mL anergic antigen; and TNF-α: positive control with a concentration of 10 ng/mL), and three test groups (CD40 mAb: 1 μg/mL; OK-432: 10 μg/mL, and 40,432 group: 1 μg/mL CD40 mAb plus 10 μg/mL OK-432). FACS gating of DCs and correlative subsets was presented in Additional file 1: Fig. S1. DCs are a population of Lin^−^HLA-DR^+^ cells. We firstly detected the expression of HLA-DR to group them into the subtype of DCs. The agent-treated groups express HLA-DR, which were uniform with the control group with marginal increasement (Fig. 1A). CD83, as a member of the immunoglobulin superfamily, is usually expressed on membrane or in soluble forms. It can regulate cell maturation and activation, the emerging of which can be a promising immune modulator with therapeutic potential. Expression of membrane CD83 can be detected on diverse activated immune cells. Mature DCs are one of the immune cells that most express CD83 highly and stably [55, 56]. We measured the expression of CD83 to identify the maturity of human DCs [55], and found that the expression of CD83 was increased by both CD40 Ab and OK-432, and their combination further enhanced the CD83 expression (Fig. 1B). Additionally, an increased ratio of type 1 DCs was detected (Fig. 1C), suggesting an enhanced potential of inducing the generation of CTLs [57]. More interestingly, the combination of CD40 Ab and OK-432 upregulated the production of IL-12, which was not detected in the single-agent-treated group (Fig. 1D). IL-12 plays a critical role in both innate and adaptive antitumor immunity. IL-12 production by DCs can augment Th1 response and stimulate the differentiation and lytic capacity of CTLs [58].

### DCs induced by CD40 Ab and OK-432 promoted the proliferation of cytotoxic T cells while suppressing Treg cells

**Fig. 2:**
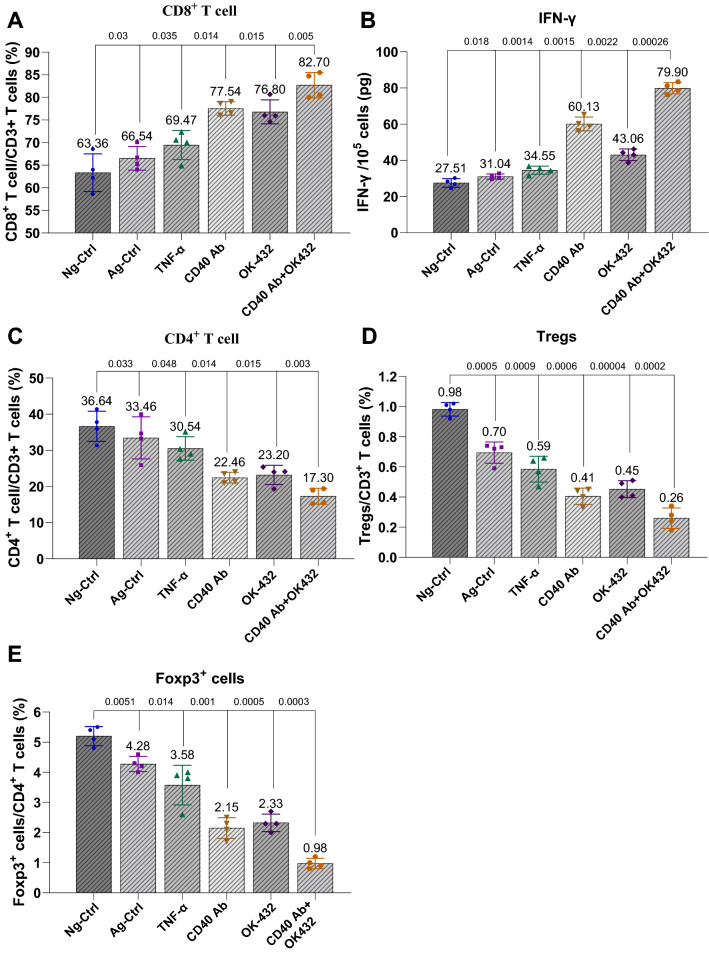
DCs induced by CD40 Ab and OK-432 promoted the proliferation of cytotoxic T cells while suppressing the number of Tregs. **A** DCs induced by CD40 Ab and OK-432 stimulated the generation of cytotoxic T cells. T cells derived from peripheral blood were treated with different types of DCs induced by relative agents. The combinational group showed relatively more CD8^+^ cells. **B** DCs induced by CD40 Ab and OK-432 augmented the secretion of IFN-γ. The three agents facilitated the production of stimulatory cytokine IFN-γ. The combinational group displayed an obvious advantage. **C** Combination of CD40 Ab and OK-432 reduced the number of CD4^+^T cells. Compared with the control groups, the three test groups had a reduced ratio of CD3^+^CD4.^+^ T cells versus total T cells. **D** DCs induced by CD40 Ab and OK-432 restrained the production of Tregs. The proliferation of Tregs originating from peripheral blood was suppressed in the three test groups, especially the combinational group. **E** DCs induced by CD40 Ab and OK-432 suppressed the expression of Foxp3. The expression of Foxp3 was obviously suppressed in the three test groups; the effect was significant in the combinational group. Value represents mean ± standard error of mean. P-value represents the comparison of control group versus tested groups

Given the positive role of CD40 Ab and OK-432 in human DC activation, we next investigated the potential effect of DCs on the generation of CTLs. We observed that human DCs induced by CD40 Ab or OK-432 could induce the generation of cytotoxic T cells (Fig. 2A). The combination of CD40 Ab with OK-432 could generate more CD8^+^ cells compared with their independent treatment. Moreover, a higher level of IFN-γ was produced using the combination (Fig. 2B), suggesting a stronger antitumor potential.

Meanwhile, we also found that the combination of CD40 Ab and OK-432 reduced the ratio of CD3 + CD4 + T cells versus total T cells (Fig. 2C), which otherwise indicated an increase in the number of CTLs. Treg cells are opposite to the stimulation of T cells. Our data showed that the proliferation of Treg cells originating from peripheral blood was suppressed by CD40 Ab and OK-432, especially in the combination group (Fig. 2D), which was verified by the expression of Foxp3 (Fig. 2E), a specific marker of Tregs.

Collectively, these results demonstrated that the combination of CD40 Ab and OK-432 boosted the maturation of not only mouse DCs but also human DCs. Both could stimulate CTLs, providing a preclinical potential against colorectal cancer.

### CD40 Ab and OK-432 played synergistic roles in activating DCs

DCs were defined by the expression of costimulatory signals CD80 and CD86 (ligands of CD28 expressed on T cells). We identified the CD8α^+^CD11c^+^CD11b^−^ subset of DCs (Additional file 1: Fig. S2), which was committed to play a critical role in CD8^+^ T cell activation [59, 60]. The cytokines and corresponding agents were detected to assess the immune response to CD40 mAb and OK-432 combination (termed as 40–432).

**Fig. 3:**
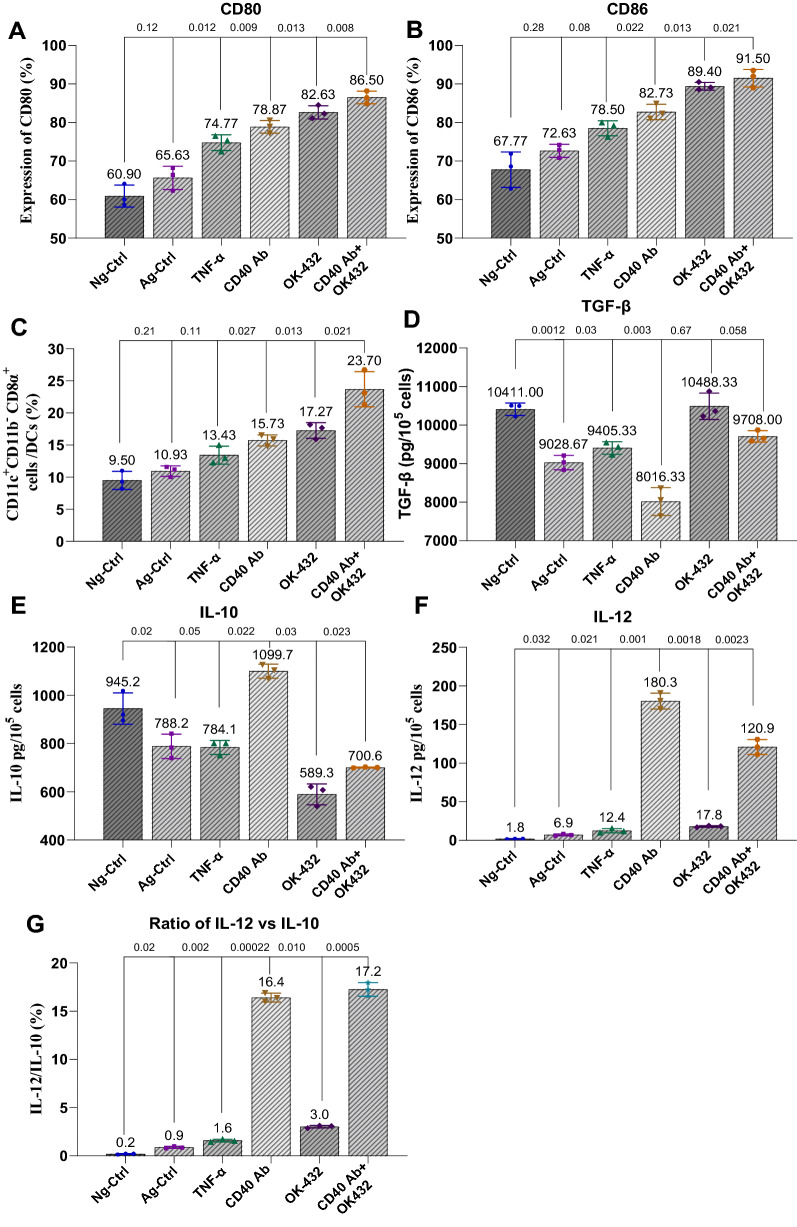
CD40 Ab and OK-432 showed synergistic action in activating mouse DCs.** A** CD40 Ab and OK-432 synergistically activated the expression of CD80. The experiment was performed in six groups, including three control groups and three test groups. Compared with the control groups (Neg-Ctrl, Ag-Ctrl, and TNF-α), the expression of CD80 was separately improved by CD40 Ab and OK-432; the improvement in the synergistic group was most significant. **B** CD40 mAb and OK-432 synergistically activated the expression of CD86. The expression of CD86 showed the same tendency as CD80 in (**A**). **C** CD40 mAb and OK-432 activated the population of CD11c^+^CD11b^−^CD8^+^ subset of DCs. The synergistic group (CD40 Ab + OK-432) showed a significant improvement in the number of CD11c^+^CD11b^−^CD8^+^ DCs. **D** Combination of CD40 Ab and OK-432 modestly affected the secretion of TGF-β. Combination of CD40 Ab and OK-432 affected the secretion of TGF-β. Value represents mean ± standard error of mean. ^*^P < 0.05, ^**^P < 0.01. **E** Combination of CD40 Ab and OK-432 suppress the secretion of IL-10. **F** Combination of CD40 Ab and OK-432 improve the secretion of IL-12. **G** Combination of CD40 Ab and OK-432 improve the ratio of IL-12/IL-10. The combinational group boosts the secretion of IL-12 and suppresses IL-10. Value represents mean ± standard error of mean. P-value represents the comparison of control group versus tested groups

The combination of CD40 mAb or OK-432 showed a stronger effect in terms of stimulating the expression of CD80 (Fig. 3A) and CD86 (Fig. 3B) compared with the single-agent-treated groups. Besides, 40–432 significantly stimulated the generation of CD11c^+^CD11b^−^CD8α^+^ subset of DCs, a subset especially contributing to the stimulation of CD8^+^ T cells, which was superior to the other agents (Fig. 3C). We observed that the combined 40–432 modestly affected the secretion of inhibitory cytokine TGF-β, which was better than OK-432 (Fig. 3D). The stimulatory cytokine IL-12 played a critical role in both innate and adaptive antitumor immunity [58]. In contrast, the level of inhibitory cytokine IL-10 was altered after stimulation with OK-432. The combined 40–432 was found to upregulate IL-12 and downregulate the level of IL-10 (Fig. 3E–G), indicating the advantage of combination.

### DCs induced by CD40 mAb and OK-432 conferred T cells with enhanced killing efficiency

**Fig. 4:**
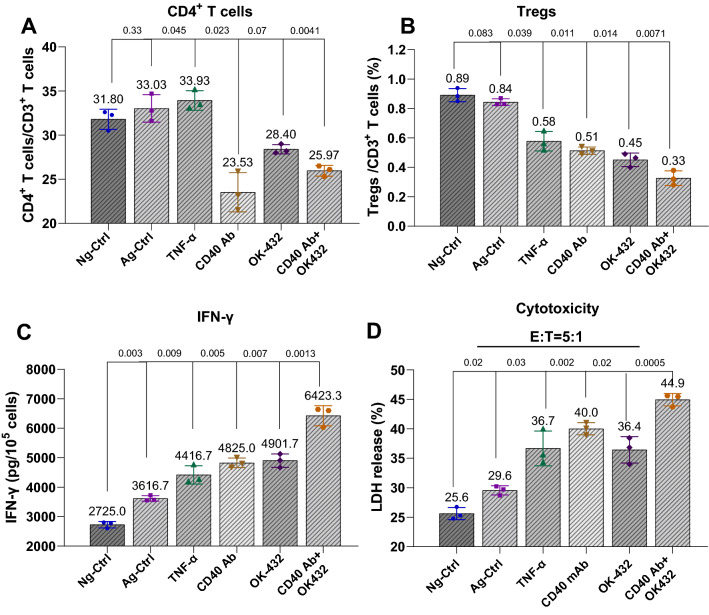
DCs induced by the combination of CD40 Ab and OK-432 enhanced the killing efficiency of T cells. **A** Combination of CD40 Ab and OK-432 reduced the number of CD4^+^T cells. The experiment was designed as six groups, including three control groups: Neg-Ctrl (without any agent), Ag-Ctrl (with anergic antigen), and TNF-α (positive control), and three test groups: CD40 Ab, OK-432, and 40,432 groups (combinational group, CD40 Ab, and OK-432). The cells were separately treated in the six groups, and the number analysis of T cells was performed using FCM. Compared with the control groups, the three test groups showed a significant reduction in the ratio of CD3^+^CD4^+^ T cells versus total T cells. **B** Combination of CD40 Ab and OK-432 reduced the number of Tregs. The test groups showed the suppression of the generation of inhibitory cells Tregs. **C** Combination of CD40 Ab and OK-432 promoted the secretion of IFN-γ. The test groups showed the production of IFN-γ from T cells. **D** Combination of CD40 Ab and OK-432 displayed high cytotoxicity to CT26 cells. The cancer cells were treated with stimulated T cells at an effector: target (E:T) ratios of 5:1. The higher-ratio group showed higher killing efficiency. In general, the test groups showed stronger cytotoxicity. Value represents mean ± standard error of mean. P-value represents the comparison of control group versus tested groups

Given the positive role of CD40 mAb and OK-432 in DC activation, we next investigated the potential effect of DCs on cytotoxic T cells (CTLs). FACS gating of T cells and correlative subsets were presented in Additional file 1: Fig. S3. CTLs used in the experiment were prepared via the method of ‘preparation of CTLs’. T cells were first separately co-cultured with DCs treated with different agents. We observed that CD40 mAb and OK-432, alone or in combination, could reduce the ratio of CD3^+^CD4^+^ T helper cells (Fig. 4A), indicating a reciprocal relationship between CD4^+^ and CD8^+^ cells. We found that the combination restrained the generation of inhibitory cells Tregs (CD4^+^CD25^+^Foxp3^+^), indicating a superior role of the combination compared with the other ones (Fig. 4B). Functionally, the production of IFN-γ derived from T cells increased on treatment with CD40 mAb or OK-432, whereas even a higher level of IFN-γ was detected in the combination group (Fig. 4C). We then tested the cytotoxicity of CTLs induced by CD40 mAb and OK-432 on the CT26 CRC cell line. At an effector:target (E:T) ratio of 5:1, CTLs induced by the combination of CD40 mAb and OK-432 showed twofold enhancement of cytotoxicity against CT26 cells compared with any single agents (Fig. 4D).

### CTLs induced by DCs treated with CD40 mAb and OK-432 showed potent feasibility in treating of colon cancer model with pulmonary metastasis

**Fig. 5:**
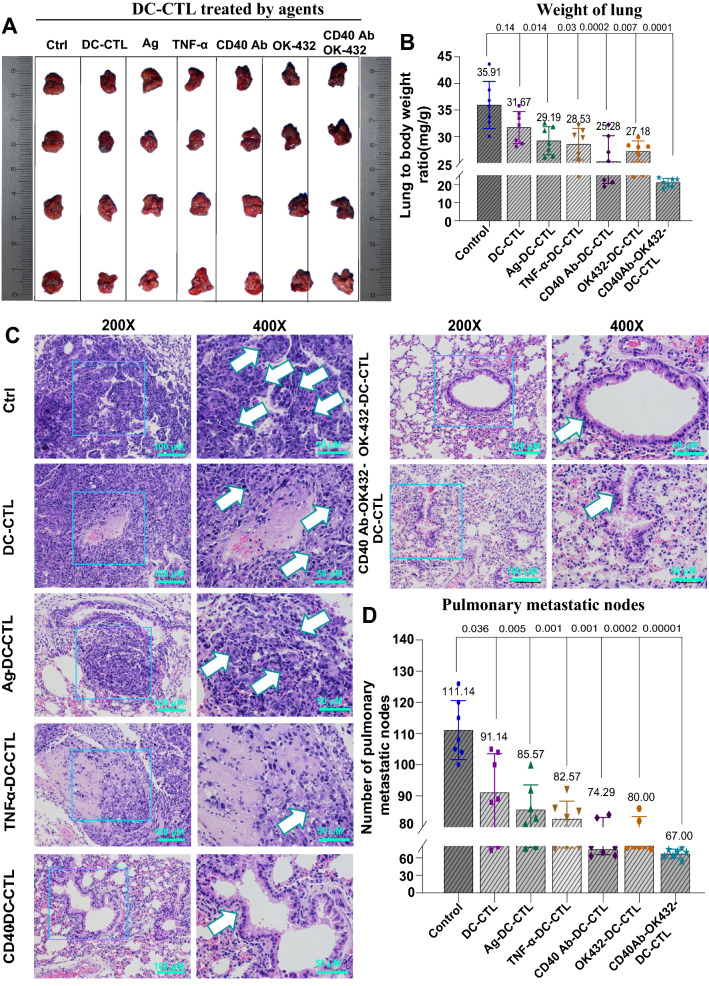
CTLs induced by CD40 Ab and OK-432 augmented the killing efficiency of tumors on lungs in colon cancer models with pulmonary metastasis. **A** Lungs derived from mouse models treated with different types of CTLs. The mice involved in this experiment were divided into seven groups. All CTLs administered to colon cancer models with pulmonary metastasis were induced by corresponding DCs. They could suppress the expansion of lungs in the mouse model. DCs activated by CD40 Ab and OK-432 elicited the most efficient CTLs, which further restrained the expansion of lungs compared with individual agents. **B** Statistics of the lung to body weight ratio (mg/g) corresponding to A. **C** H&E staining of lung slides. H&E staining was used to observe the lung morphology. Each group was presented with a magnified image. The combinational group showed the least number of tumor cells compared with the others. **D** Statistics of tumor nodes corresponding to C. Value represents mean ± standard error of mean. P-value represents the comparison of control group versus tested groups

Based on the achievement of functional CTLs, we next tested the possible role of CTLs in treating a CRC mouse model with pulmonary metastasis. The mice in this experiment were divided into seven groups. Each group was administered with corresponding CTLs originating from pertinent DCs and the administration of control group was replaced with saline. Briefly, the mice were administered with 1 × 10^7^ CTLs twice a week via tail vein injection, which was maintained for four courses. The morphology and weight of the mouse lungs were assessed to compare the tumor suppression and metastasis control efficiency of CTLs induced by different agents. We observed that the combination of CD40 mAb with OK-432 outperformed single agents in restraining the pulmonary metastasis (Fig. 5A). Both the ratio of lung weight/body weight (Fig. 5B) and the number of pulmonary metastatic nodes (Fig. 5D) delineated the most in the 40–432 group. Furthermore, H&E staining of the lung indicated fewer neoplastic cells in the 40–432 group compared with the others (Fig. 5C). Thus, CTLs generated by CD40 mAb-OK-432-induced DCs had a better antitumor effect in controlling CRC lung metastasis.

### DCs induced by CD40 mAb and OK-432 contributed to the proliferation of CTLs while facilitating the suppression of Tregs in vivo

**Fig. 6:**
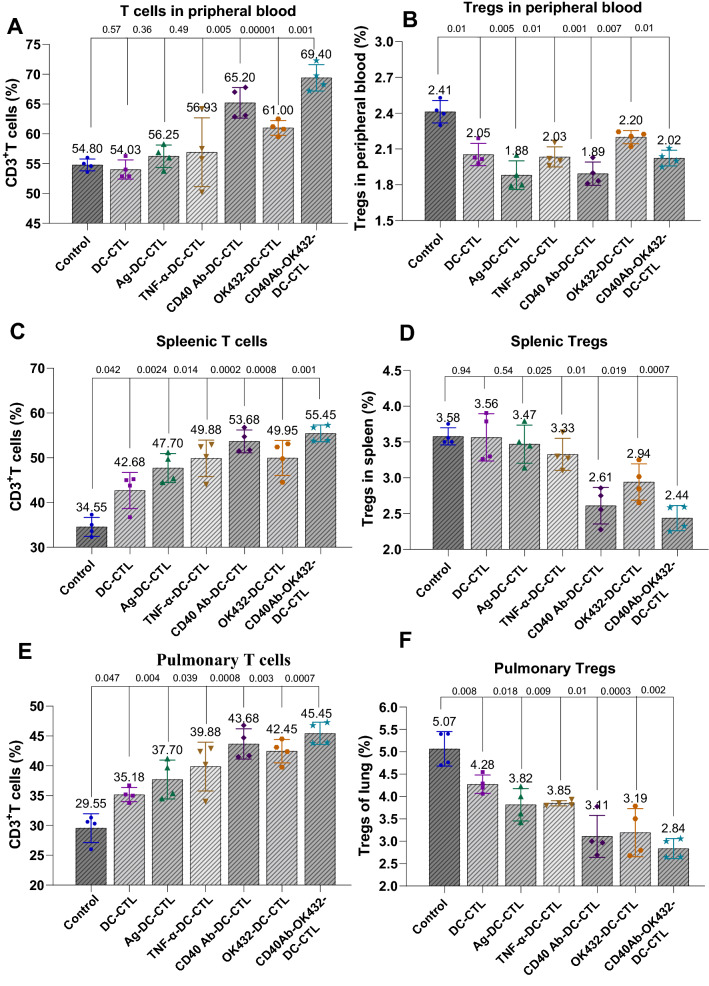
DCs induced by CD40 Ab and OK-432 activated the proliferation of cytotoxic T cells while suppressing the number of Tregs. **A** DCs induced by CD40 Ab and OK-432 stimulated the generation of cytotoxic T cells. T cells derived from three different sources (peripheral blood, spleen, and lung) were separately treated with different types of DCs. Compared with the control groups, the agent-treated groups showed an increasing number of CD3^+^ cells, while the combinational group displayed a significant proliferation. **B** DCs induced by CD40 Ab and OK-432 restrained the production of Tregs. The proliferation of Tregs originating from the three sources was suppressed by the four agents, especially in the combinational group. Value represents mean ± standard error of mean. P-value represents the comparison of control group versus tested groups

Considering that the combination of CD40 mAb and OK-432 was beneficial to the CRC model with pulmonary metastasis, we next investigated the potential effects of CTLs on specific organs. The cell sources were derived from three types of tissues: peripheral blood, spleen, and lung. After treatment, the number of CD3^+^ T cells increased with varying degrees of proliferation. The combined group showed a significant enhancement (Fig. 6A). In addition, the proliferation of Tregs originating from the three sources was suppressed, whereas the combinational group showed a prominent suppression (Fig. 6B).

**Fig. 7:**
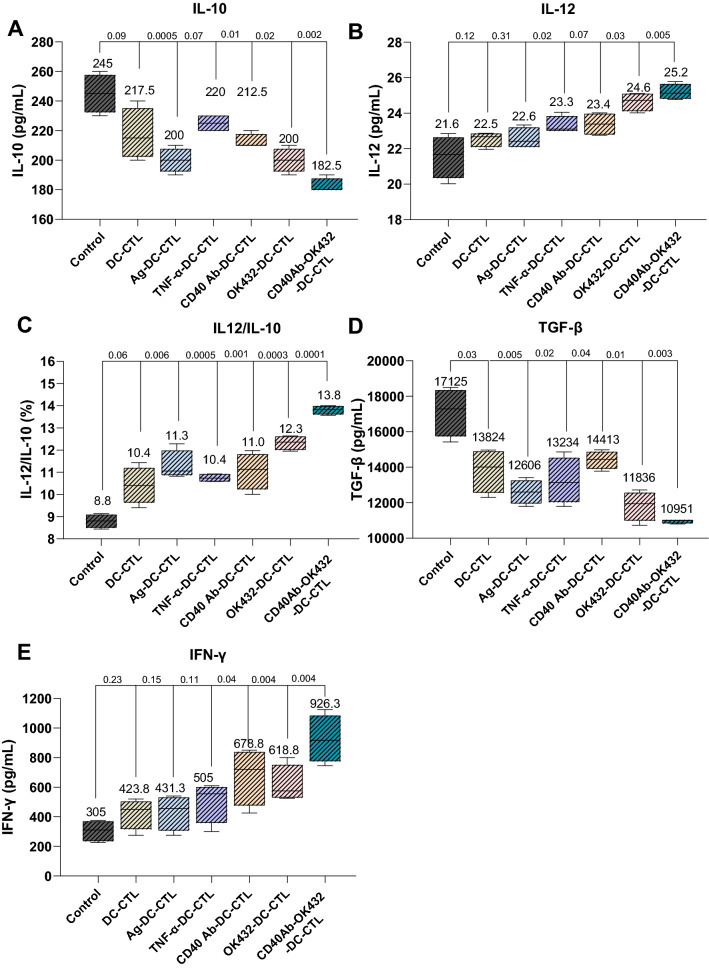
CD40 Ab and OK-432 synergistically enhanced the secretion of stimulatory cytokines while inhibiting the secretion of inhibitory cytokines. **A** CTLs induced by CD40 Ab and OK-432 inhibited the production of IL-10. The inhibitory cytokine IL-10 was restrained in the agent-treated groups, while the combinational group showed a prominent suppression. **B** CTLs induced by CD40 Ab and OK-432 inhibited the production of TGF-β. The secretion of inhibitory cytokine TGF-β was blocked in the agent-treated groups; the combinational group showed a significant change. **C** CTLs induced by CD40 Ab and OK-432 improved the ratio of IL-12/IL-10. **D** CTLs induced by CD40 Ab and OK-432 augmented the generation of IL-12. Although the four agent-treated groups contributed to the enhancement of stimulatory cytokine IL-12, the combinational group exhibited an outstanding contribution. **E** CTLs induced by CD40 Ab and OK-432 augmented the secretion of IFN-γ. The four agents facilitated the increase in the production of stimulatory cytokine IFN-γ; the combinational group displayed an obvious advantage. Value represents mean ± standard error of mean. P-value represents the comparison of control group versus tested groups

The cytokines involved in the stimulation of CTLs were also detected in peripheral blood. We observed that the production of inhibitory cytokines IL-10 and TGF-β was restrained by the agents; the combinational group contributed a significant change (Fig. 7A and B). Meanwhile, the stimulatory cytokines IL-10, IL-12 and IFN-γ were also detected. The results demonstrated that the four agents enhanced stimulatory cytokines IL-12 and IFN-γ, whereas the combined group exhibited an outstanding function (Fig. 7C–E).

## Discussion

The clinical outcomes against various cancers have improved with the development of advanced cytotoxic agents and molecular-targeted treatments. However, statistics on overall survival of such colon cancer is disappointed, because patients usually suffer from advanced colon cancer with pulmonary metastasis [61–63]. Fortunately, immunotherapy may be endued with prominent potential against such types of cancer. As APCs, DCs act as a vital component in the immune system. They can present the tumor-associated antigens to cytotoxic cells, which release cytokines targeting tumor cells. Additionally, DCs also control the immune tolerance and immunity [16, 18]. Thus, targeting DCs is a feasible strategy for cancer immunotherapy.

The immune components in a tumor microenvironment (TME) are crucial for immunotherapy [64]. Regulatory T cells (Tregs) display a vital role in suppressing the activity of CTLs, leading to immune escape and treatment failure. The increasing number of infiltrated Tregs generally indicates a poor prognosis [65, 66]. Among the CD4^+^ T cells, approximately 5%–10% of Tregs secrete cytokines interleukin-10 (IL-10) and transforming growth factor β (TGF-β) [67]. Under normal conditions, Tregs maintain immune homeostasis and induce immune tolerance. Abnormally, the dysregulation of Tregs is associated with the induction of immune tolerance, contributing to the escape of tumor cells from being attacked by immune cells. Mounting evidence has demonstrated that Treg-mediated immunosuppression is the major cause leading to the failure of tumor treatment [68, 69]. In contrast, CTLs serve a contrary role to Tregs. They secrete IFN-γ that facilitates the killing. CD80 and CD86 are involved in DC licensing and CTL activation. DCs have the MHC II (MHC class II)-peptide, which can interact with receptors on T helper cells (Th). The upregulation of CD80 and CD86 license the DCs to interact with CD8^+^ T cells [16, 70]. Therefore, as a precondition, the activation of DCs is indispensable for the stimulation of CTLs. The enhancement of DC activation certainly facilitates the generation of CTLs. One of the feasible approaches is to utilize the CD40 agonists, which can activate the DCs and promote the secretion of IL-12, furthermore turn the “cold” tumor to “hot” one [38]. However, the single-agent antitumor activity of CD40 agonists is not enough. They usually combine with other adjuvants.

Based on previous studies, we found that OK-432, as an adjuvant, was beneficial to the treatment of colon cancer via the CD40/CD40L axis [39, 42]. This project was then yielded by the hypothesis that the combination of CD40 agonists and OK-432 may confer a synergistic function, which has been demonstrated in this study. However, there are further puzzles to be investigated. We observed that the combinational group (CD40 mAb and OK-432) endowed DCs with a stronger potential to stimulate T cells and prominent cytotoxicity to tumor cells. Besides, the combination facilitated the expression of the costimulatory signals (CD80 and CD86) involved in activating DCs and the production of stimulatory cytokines (IL-12 and IFN-γ) involved in stimulating T cells. Meanwhile, it also suppressed the generation of Tregs and inhibitory cytokines (IL-10 and TGF-β). Hence, the combination of CD40 mAb and OK-432 provided a promising strategy to facilitate immunotherapy. However, several beneficial indicators associated with activated DCs or CTLs were not significantly changed, which requires further exploration and modification.

Furthermore, OK-432 has been reported to inhibit the cell proliferation via activating tumoricidal macrophages to yield cytotoxic factors in bladder cancer [71], indicating a synergistic function between different types of immune cells. That is to say, the synergistic effect of OK-432 and CD40 mAb not only avail to DCs activation but also macrophages, which is deserving of further exploration.

Presently, there is a debatable question whether and how FAS activation and deactivation affect the number of DCs. It is reasonable that antigen-specific T cells may potentially induce the apoptosis of DCs through Fas- and perforin-dependent manners. However, we did not detect the correlation between DCs number and FAS activation in the present study. We also found that there is a controversial possibility. The study reported that human CD34^+^-derived DCs and mouse DCs with either immature or mature state, are not susceptible to Fas-induced cell death. This resistance is correlated with the constitutive expression of the Fas-associated death domain-like IL-1beta-converting enzyme (FLICE)-inhibitory protein (FLIP) ligand. Their study demonstrated that Fas-activated DCs by FasL or anti-Fas antibodies can upregulate the expression of MHC II, B7, and DC-LAMP molecules. Additionally, mature DCs, if exposed to FasL, produce even higher amounts of IL-1beta [72]. Therefore, such debatable question may need further discussion and exploration.

Additionally, there still exist limitations in this study, which should be paid attention to and be explored in subsequent research. For instance, it is a better approach to employ CompuSyn software [73] to analyze the synergistic function of CD40 Ab and OK-432 at the earlier stage of study. The combinational function of OK-432 and CD40 Ab affect the stimulatory molecules rather than the significant change of inhibitory cytokine TGF-β. It is the deficiency of present combination, which need to be replenished in the subsequent study. Applicable inhibitors of CD40 Ab should have been found and applied into the study to specifically demonstrate the synergistic function of CD40 Ab and OK-432. Subsequent study should be extended to investigate the combinational function in vivo.

## Conclusions

This study demonstrated that the combination of CD40-mAb and OK-432 promoted the maturation of DCs, augmented the production of stimulatory cytokines, and suppressed the secretion of inhibitory cytokines, which in turn significantly enhanced the activation of CTLs by DCs, both in vitro and in vivo. The combination of CD40-mAb and OK-432 might provide a promising approach for clinical treatment against CRC.

## Supplementary Information

**Additional file 1: Table S1.** Agents used in the FCM. **Figure S1.** FACS gating of DCs and correlative subsets. The FACS gating strategy was listed as following: first, DCs were gated as Gate 1; then, CD80^+^/CD86^+^ cells were gated as Gate 2, which was gathered in the lower-right quadrant. The correlative subsets were gated with the similar strategy. DCs were gated as Gate 1; then, CD11c^+^ cells were gated as Gate 2, CD8a^+^ cells were gated as Gate 3, and CD11b^+^ cells were gated as Gate4. **Figure S2.** CD40 Ab and OK-432 synergistically activate the expression of CD80. A, B. Identification of dendritic cells (DCs). DC-associated markers (CD8α, CD80, CD86, CD11c, and CD11b) were employed to identify the subset of DCs, which was achieved by FCM. DCs marked by the co-stimulatory signals (CD80, CD86) are more than 85%. 36.7% were marked by DCs defining marker CD11c, among of which 12.9% were CD11b-marked cells. And these markers defined the DC subset that is committed to play critical role in T cell activation. C, D. CD40 Ab and OK-432 synergistically activate the expression of CD80. The experiment was performed in six groups, including three control groups: Neg-Ctrl (without any agent), Ag-Ctrl (with anergic antigen), and TNF-α (positive control), and three test groups: CD40 Ab, OK-432, and 40432 group (combinational group, CD40 Ab and OK-432). Compared to the control groups (Neg-Ctrl, Ag-Ctrl, and TNF-α), expression of CD80 can be separately improved by CD40 Ab, and OK-432, and the synergistic group 40432 was most significantly improved. **Figure S3. **FACS gating of T cells and correlative subsets. The FACS gating strategy was listed as following: first, T cells were gated as Gate 1; then, CD8^+^ cells were gated as Gate 2. CD4^+^ cells were gated as Gate 2, Foxp3^+^ cells were gated as Gate 3.

## Data Availability

All data generated or analyzed in this study are included in this published manuscript.
