# Supplementary material for: CD40 monoclonal antibody and OK432 synergistically promote the activation of dendritic cells in immunotherapy
Source: Cancer Cell Int. 2022 Jun 17;22:216. doi: 10.1186/s12935-022-02630-x (PMC9206283; doi:10.1186/s12935-022-02630-x)
Supplement: Supplementary file 1 — Additional file 1: Table S1. Agents used in the FCM. Figure S1. FACS gating of DCs and correlative subsets. The FACS gating strategy was listed as following: first, DCs were gated as Gate 1; then, CD80+/CD86+ cells were gated as Gate 2, which was gathered in the lower-right quadrant. The correlative subsets were gated with the similar strategy. DCs were gated as Gate 1; then, CD11c+ cells were gated as Gate 2, CD8a+ cells were gated as Gate 3, and CD11b+ cells were gated as Gate4. Figure S2. CD40 Ab and OK-432 synergistically activate the expression of CD80. A, B. Identification of dendritic cells (DCs). DC-associated markers (CD8α, CD80, CD86, CD11c, and CD11b) were employed to identify the subset of DCs, which was achieved by FCM. DCs marked by the co-stimulatory signals (CD80, CD86) are more than 85%. 36.7% were marked by DCs defining marker CD11c, among of which 12.9% were CD11b-marked cells. And these markers defined the DC subset that is committed to play critical role in T cell activation. C, D. CD40 Ab and OK-432 synergistically activate the expression of CD80. The experiment was performed in six groups, including three control groups: Neg-Ctrl (without any agent), Ag-Ctrl (with anergic antigen), and TNF-α (positive control), and three test groups: CD40 Ab, OK-432, and 40432 group (combinational group, CD40 Ab and OK-432). Compared to the control groups (Neg-Ctrl, Ag-Ctrl, and TNF-α), expression of CD80 can be separately improved by CD40 Ab, and OK-432, and the synergistic group 40432 was most significantly improved. Figure S3. FACS gating of T cells and correlative subsets. The FACS gating strategy was listed as following: first, T cells were gated as Gate 1; then, CD8+ cells were gated as Gate 2. CD4+ cells were gated as Gate 2, Foxp3+ cells were gated as Gate 3. [file 12935_2022_2630_MOESM1_ESM.docx]

**Supplemental tables**

**Table S1: Agents used in the FCM**

| **Reagents** | **Company** | **Product #** |
| --- | --- | --- |
| CD8a--PerCp | Miltenyi Biotec, Germany | 53-6.7/130-122-953 |
| CD80--FITC | Miltenyi Biotec, Germany | 16-10A1/130-102-882 |
| CD86--PE | Miltenyi Biotec, Germany | PO3.3/130-123-724 |
| CD11c--FITC | Miltenyi Biotec, Germany | N418/130-122-939 |
| CD11b--PE | Miltenyi Biotec, Germany | REA592/130-113-806 |
| CD40 Ab (FGK45) | AdipoGen, USA | AG-20B-0036PF |
| OK-432 | T&L Biological Technology, China | GMP-TL107-0100 |
| IL-10 | eBioscience | BMS614-2FIVE |
| IL-12 | Biolegend, USA | 433607 |
| TGF-β | Alpco, USA | SMB100B |
| TNF-α | Biolegend, USA | 430907 |
| IFN-γ | Alpco, USA | MBS2506010 |
| CD3-APC | Miltenyi Biotec, Germany | REA641/130-122-943 |
| CD4--FITC | Miltenyi Biotec, Germany | GK1.5/130-120-819 |
| Foxp3-PE | Miltenyi Biotec, Germany | REA788/130-111-600 |


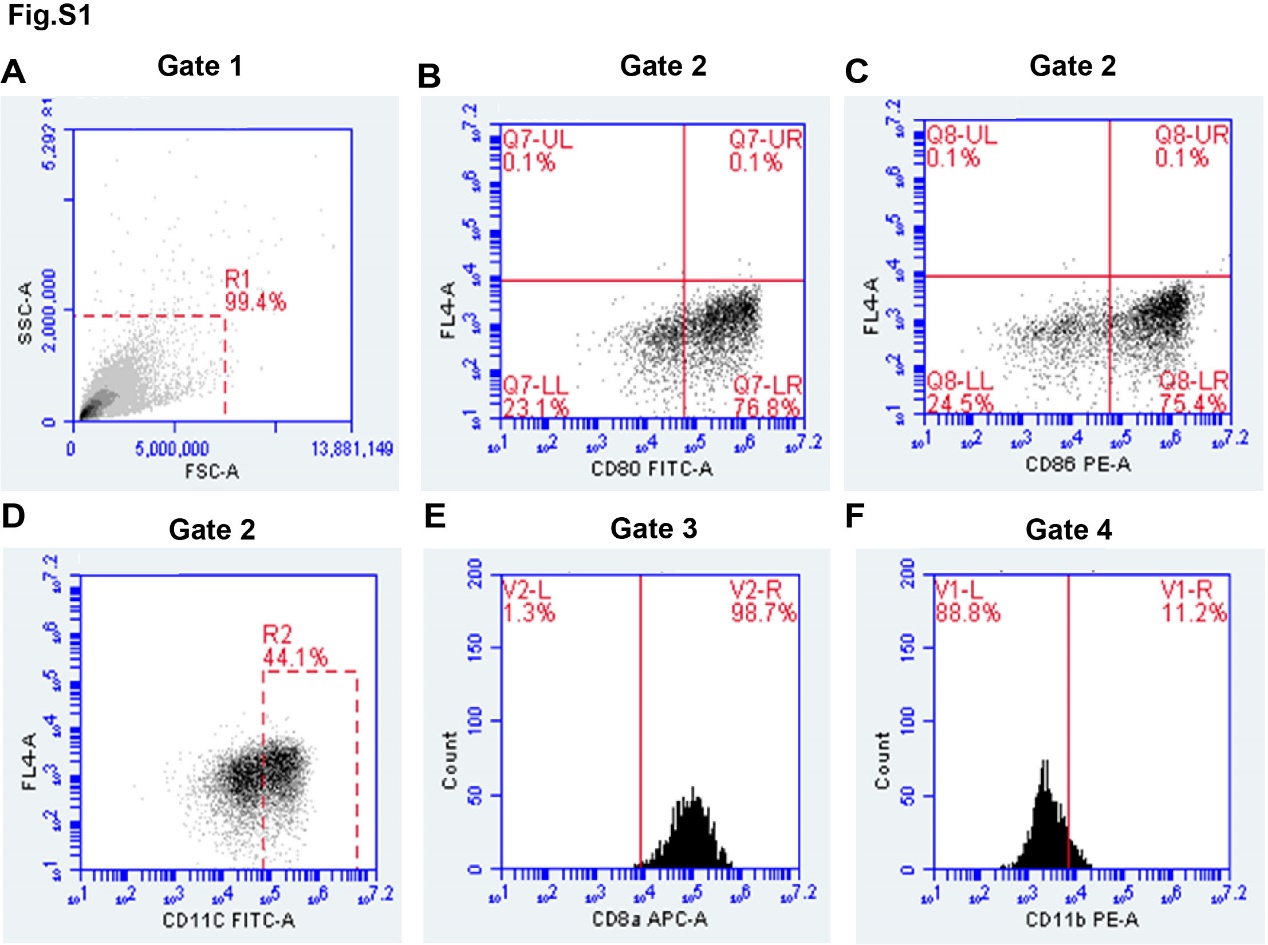


**Fig. S1** FACS gating of DCs and correlative subsets. The FACS gating strategy was listed as following: first, DCs were gated as Gate 1; then, CD80^+^ / CD86^+^ cells were gated as Gate 2, which was gathered in the lower-right quadrant. The correlative subsets were gated with the similar strategy. DCs were gated as Gate 1; then, CD11c^+^ cells were gated as Gate 2, CD8a^+^ cells were gated as Gate 3, and CD11b^+^ cells were gated as Gate4.


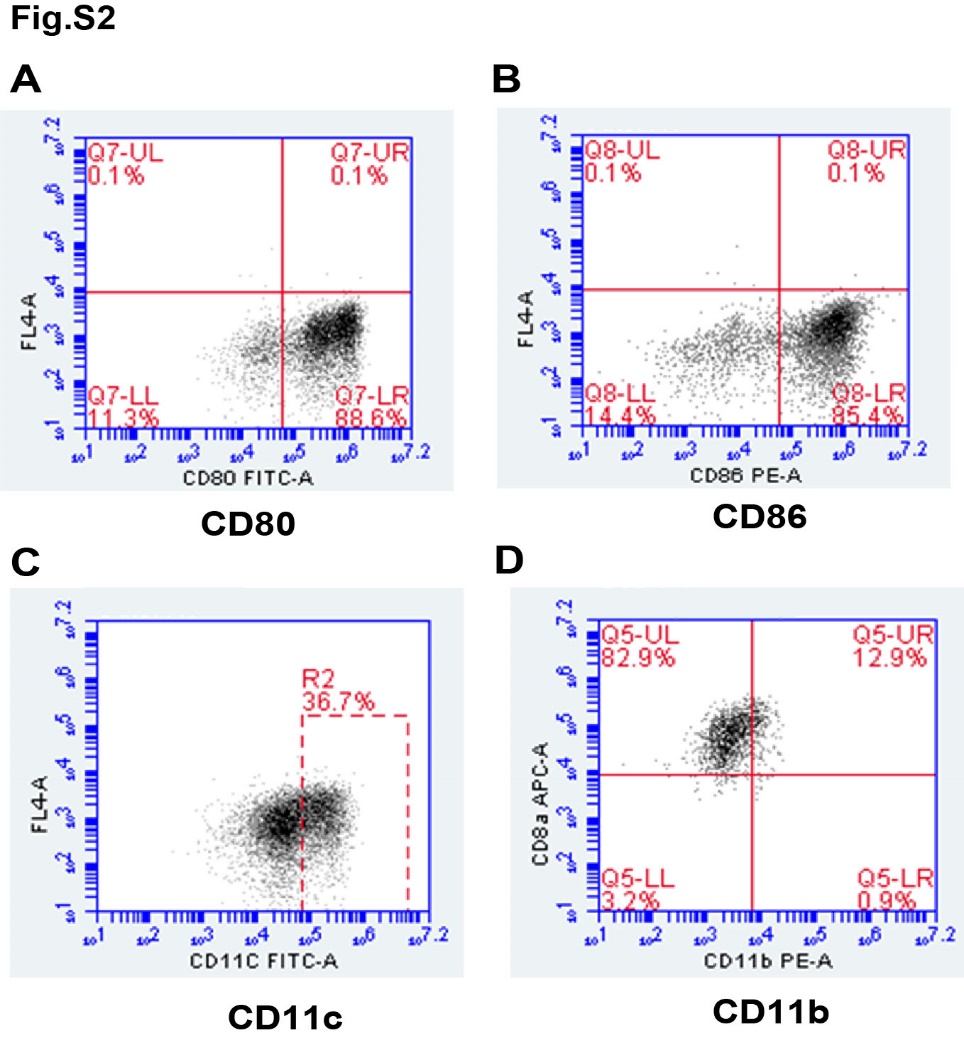


**Fig.S2** CD40 Ab and OK-432 synergistically activate the expression of CD80.

A, B. Identification of dendritic cells (DCs). DC-associated markers (CD8α, CD80, CD86, CD11c, and CD11b) were employed to identify the subset of DCs, which was achieved by FCM. DCs marked by the co-stimulatory signals (CD80, CD86) are more than 85%. 36.7% were marked by DCs defining marker CD11c, among of which 12.9% were CD11b-marked cells. And these markers defined the DC subset that is committed to play critical role in T cell activation.

C, D. CD40 Ab and OK-432 synergistically activate the expression of CD80. The experiment was performed in six groups, including three control groups: Neg-Ctrl (without any agent), Ag-Ctrl (with anergic antigen), and TNF-α (positive control), and three test groups: CD40 Ab, OK-432, and 40432 group (combinational group, CD40 Ab and OK-432). Compared to the control groups (Neg-Ctrl, Ag-Ctrl, and TNF-α), expression of CD80 can be separately improved by CD40 Ab, and OK-432, and the synergistic group 40432 was most significantly improved.


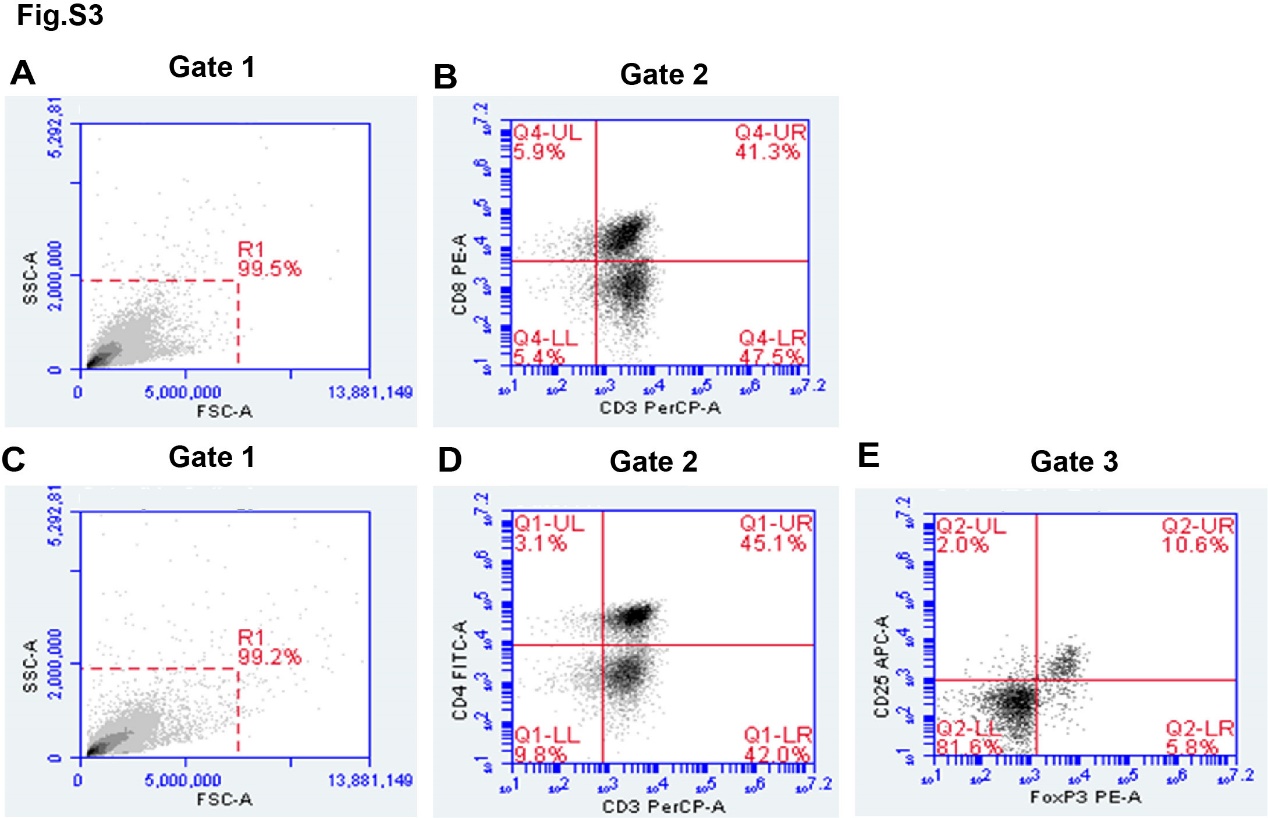


**Fig.S3** FACS gating of T cells and correlative subsets. The FACS gating strategy was listed as following: first, T cells were gated as Gate 1; then, CD8^+^ cells were gated as Gate 2. CD4^+^ cells were gated as Gate 2, Foxp3^+^ cells were gated as Gate 3.
